# Systematic Design of Trypsin Cleavage Site Mutated Exendin4-Cysteine 1, an Orally Bioavailable Glucagon-Like Peptide-1 Receptor Agonist

**DOI:** 10.3390/ijms18030578

**Published:** 2017-03-08

**Authors:** Wenbo Sai, Hong Tian, Kangmin Yang, Daoqi Tang, Jinxiao Bao, Yang Ge, Xiaoda Song, Yu Zhang, Cheng Luo, Xiangdong Gao, Wenbing Yao

**Affiliations:** Jiangsu Key Laboratory of Druggability of Biopharmaceuticals, School of Life Science and Technology, China Pharmaceutical University, Nanjing 210009, China; wenbosai@hotmail.com (W.S.); tinahew@139.com (H.T.); ykmylx@126.com (K.Y.); yitangchen@aliyun.com (D.T.); m15295783798@163.com (J.B.); 18251380528@163.com (Y.G.); xiaoda_song@126.com (X.S.); zhangyu2007sm@163.com (Y.Z.); luorogen@163.com (C.L.)

**Keywords:** exednin-4, exendin4-cysteine, orally administered, TSME-1, type 2 diabetes

## Abstract

Exendin-4 is a strong therapeutic candidate for the treatment of metabolic syndrome. Related receptor agonist drugs have been on the market since 2005. However, technical limitations and the pain caused by subcutaneous injection have severely limited patient compliance. The goal of the study is to investigate a biologically active exendin-4 analog could be administered orally. Using intraperitoneal glucose tolerance tests, we discovered that exendin4-cysteine administered by oral gavage had a distinct hypoglycemic effect in C57BL/6J mice. Using Rosetta Design and Amber, we designed and screened a series of exendin4-cysteine analogs to identify those that retained biological activity while resisting trypsin digestion. Trypsin Cleavage Site Mutated Exendin4-cysteine 1 (TSME-1), an analog whose bioactivity was similar to exendin-4 and was almost completely resistant to trypsin, was screened out. In addition, TSME-1 significantly normalized the blood glucose levels and the availability of TSME-1 was significantly higher than that of exendin-4 and exendin4-cysteine. Collectively orally administered TSME-1, a trypsin-resistant exendin-4 analog obtained by the system, is a strong candidate for future treatments of type 2 diabetes.

## 1. Introduction

Metabolic disorders, including type 2 diabetes (T2D), obesity, hyperlipidemia, and hypertension, affect millions of people worldwide. Currently, the treatments for these disorders are limited. Available medications are frequently associated with poor tolerability and patient compliance, thus resulting in sub-optimal outcomes [1,2]. The need for additional drugs or alternative treatments that are easily administered has become an urgent issue in biomedical research. Glucagon like peptide-1 (GLP-1) is an incretin secreted by the L cells of the intestine. Since its identification by Nauck in 1986 [3], increasing evidence has demonstrated various biological functions of GLP-1 through the activation of the glucagon-like peptide-1 receptor (GLP-1R). These roles include improving insulin biosynthesis and secretion, inhibiting glucagon secretion, suppressing appetite, and increasing the insulin sensitivity of the liver and peripheral tissues [4]. Advances in the research of GLP-1 have provided a new approach to the treatment of metabolic disorder-related symptoms [5,6,7]. Unlike other drugs, GLP-1R agonists also promote β cell proliferation and inhibit β cell apoptosis [8,9]. Since 2005, Bydureon^®^, Victoza^®^, Eperzan^®^ and other GLP-1 analog drugs have joined an extensive list of existing drugs, thus indicating the importance and favorable research prospects of related candidates [10,11,12].

However, the administration route of subcutaneous injection (SI) continues to be an unavoidable problem for these medications. This route requires skilled hands and portable devices, and can be quite painful. These limitations negatively affect patient compliance. A 6-month follow-up study has reported that the percentage of patients receiving liraglutide and exenatide decreased to 80% after one month and to 65% after six months [13]. This study has emphasized the need for alternative analogs that can be orally administered. In 2015, the phase II clinical trial data for Semaglutide (Drug Code: OG217SC, Novo Nordisk, Bagsvaerd, Denmark), an oral GLP-1 analog, became available and demonstrated the main expected clinical outcome for T2D treatment. From a mean baseline HbA1c of 7.9%, people treated with oral semaglutide was improved by 0.7% to 1.9% in different dose groups after 26 weeks [14]. Thus, the oral peptide could potentially replace subcutaneous injections for treating T2D. Although there are many important advances in the field of oral biotechnology drug research, there remains a lack of successfully treated product. For Semaglutide, the most anticipated product on the market, the oral dose is approximately 70–280 times greater than that of the subcutaneous injection. The low bioavailability is primarily caused by two factors: efficient metabolism and inefficient absorption [15,16].

Researchers have tested various methods to enhance the bioavailabilities of oral hypoglycemic exendin-4 (Ex4) analogs, including biotin and transport peptide modifications, among others. However, an additional fragment may simultaneously affect the analog’s absorption. According to a previous study, the decomposition of l-cysteine into hydrogen sulfide stimulates the opening of the intestinal epithelial potassium ion channels, which may cause potassium efflux and hyperpolarization of the cell [17] and subsequently promote intestinal absorption of the nutrient. Therefore, we speculated that the l-cysteine modification on the C-terminus of Ex4 might achieve a higher bioactivity and bioavailability after oral administration.

In this study, the essence is to obtain a balance between the pharmacodynamics and pharmacokinetic values of peptide drugs, which has historically been the primary challenge and goal in biological macromolecule studies. According to the hypothesis, the cysteine added to the carboxyl terminal would significantly improve the blood control activity of Ex4 when adminstrated by oral gavage (OG). The computer aided design would be used for site-specific mutagenesis, which is in order to improve the stability of the analogs against the proteases and retain the bioactivity to the maximum extent. The direct aim of this research is to obtain an analog with good glycemic control activity taken orally.

## 2. Results

### 2.1. Modest Normalization of Blood Glucose Levels by Exendin4-Cysteine (Ex4C) in Intraperitoneal Glucose Tolerance Tests (IPGTTs)

**Figure 1:**
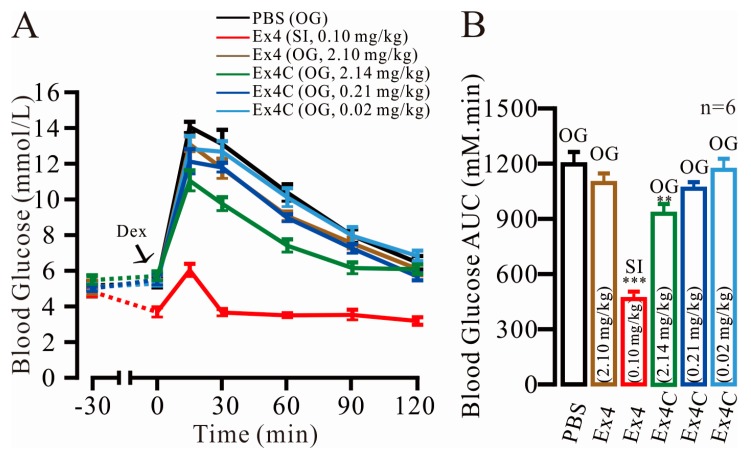
Intraperitoneal glucose tolerance test for Exendin-4 (Ex4) and Exendin4-cysteine (Ex4C) in C57BL/6J mice. (**A**) The blood glucose lowering effects of the Ex4 (subcutaneous injection) and Ex4C (subcutaneous injection/oral gavage) treatments in C57BL/6J mice; (**B**) Area under the curve of the blood glucose level during the intraperitoneal glucose tolerance tests. The data are presented as the mean ± SEM, *n* = 6, *** *p* < 0.001.

The experiment was conducted on the following six groups: Control (PBS, OG), 0.10 mg/kg (peptide mass/ mouse weight) Ex4 (SI), 2.10 mg/kg Ex4 (OG), 0.02 mg/kg Ex4C (OG), 0.21 mg/kg Ex4C (OG) and 2.14 mg/kg Ex4C (OG). The blood glucose level (BGL) time-course is shown in Figure 1A. Ex4 (OG) showed no significant improvement in BGL compared with the PBS group. At 15 min, when compared with PBS group, the BGL of 2.14 mg/kg Ex4C-traeted group was clearly suppressed (14.28 ± 0.30 vs. 11.25 ± 0.58 mM, *p* = 9.33 × 10^−4^). The Ex4C (OG) group achieved 37.45% treatment effect of Ex4 (SI). The 0.21 mg/kg Ex4C also showed an improvement in lowering the BGL in 60 min (*n* = 6, *p* < 0.05). At 120 min, the BGL of 2.14 mg/kg Ex4C (OG) became no difference with the PBS group, which possibly resulted in a protease-induced hydrolysis effect. The area under the curve (AUC), which showed the overall effect of BGL control over time, was calculated (Figure 1B). Ex4 (SI) had the strongest treatment effect, and the AUC was only 44.00% that of the control (473.88 ± 18.99 vs. 1214.67 ± 46.38, *p* = 4.03 × 10^−8^). The values of the 2.14 mg/kg Ex4 (OG) and 2.14 mg/kg Ex4C (OG) groups were 1112.00 ± 29.68 (*p* = 0.09 to the PBS group) and 945.68 ± 32.61 (*p* = 7.87 × 10^−4^ to the PBS group), respectively. For the latter group, although the results were significant, the BGL decrease was only 22.16%, despite the 20-fold increase in concentration, thus indicating that the BGL normalization ability and the duration of the effect when Ex4C is orally administered would not be satisfactory. 

### 2.2. Three Key Proteases and Seven Specific Cleavage Sites in Ex4C Were Confirmed

**Figure 2:**
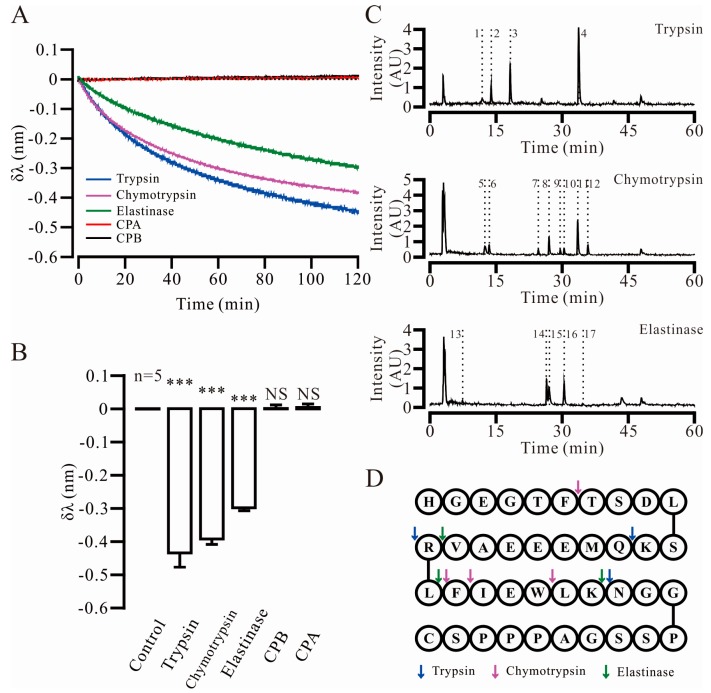
Confirmation of the key proteases and cleavage sites for Exendin4-cysteine (Ex4C) in the small intestine. (**A**) The negative migration of the reflected lights, measured as the change in wavelength in nm, indicated the changing shape of the substrate and the hydrolysis efficiency. (**B**) Bio-layer interferometry data represent five separate experiments. The data are presented as the mean ± SEM, *n* = 5, *** *p* < 0.001, NS means no significant difference. (**C**) The molecular weights of the hydrolyzed Ex4C fragments were confirmed by liquid chromatography-mass spectroscopy (LC-MS). Each number represents a fragment analyzed by MS. The analysis is provided in Table 1. (**D**) The amino acid sequence of Ex4C is shown in the figure with the letters. The seven specific cleavage sites for the three proteases were determined by analyzing the fragments and showed in different colors.

According to previous studies, the major proteases in the small intestine are secreted by the pancreas and include trypsin, chymotrypsin, elastase, carboxypeptidase-A (CPA) and carboxypeptidase-B (CPB) [18,19,20]. In Figure 2A, the negative migration of the reflected light in bio-layer interferometry assays, measured as change in wavelength in nm, which is represented by δλ and indicated the proteases hydrolysis efficiency to the substrate. After a 120-min reaction, the migration of three sensors was evident relative to the original baseline. CPA and CPB did not bind or hydrolyze the substrate, which remained unchanged relative to the baseline. The statistical results of the bio-layer interferometry (BLI) (Figure 2B) showed that trypsin (δλ = −0.47 ± 0.05 nm, *p* = 7.71 × 10^−6^), chymotrypsin (δλ = −0.39 ± 0.02 nm, *p* = 2.19 ×10^−8^) and elastase (δλ = −0.31 ± 0.01 nm, *p* = 1.31 × 10^−9^) were the key active proteases in the small intestine. To verify the accuracy of the new technology, HPLC was used to verify the data. The results of the validation are shown in supplementary Figure S1. Liquid chromatography-mass spectroscopy (LC-MS) was used to identify the specific cleavage site in Ex4C (Figure 2C). The sample was hydrolyzed by the three key proteases, and the fragments of each product were separated by liquid chromatography, as marked with “No.” in the Figure 2C. The mass spectra of the fragments were analyzed to calculate the molecular weights and assigned individual fragments based on changes in ionization. The result was listed in Table 1. The analysis identified the amino acids of Lys^12^, Arg^20^, Lys^27^ as the trypsin cleavage sites, Phe^6^, Leu^21^, Phe^22^, Trp^25^ as the chymotrypsin cleavage sites, and Val^19^, Leu^21^, Lys^27^ as the elastase cleavage sites. The cleavage sites are indicated in Figure 2D with colored arrows.

### 2.3. Trypsin Cleavage Site Mutated Exendin4-Cysteine 1 (TSME-1) Was Designed and Screened under the Assistance of Computer

Because trypsin is the predominant protease in the small intestine, trypsin-resistance was given priority in the computer-based design. Table 2 shows the stability of each specific amino acid mutation in the trypsin cleavage site predicted by the Rosetta Design Server; a lower score indicates a more stable mutation. For the 3 sites of predicted cleavage, three mutations per site were predicted to form stable structures, and the predicted differences in binding scores were small. This data predicted that we could successfully design in substitutions to the Ex4C sequence that would be resistant to trypsin degradation while retaining high binding affinity. Eight analogs were designed through site-specific mutations, permutations and combinations. These analogs were modeled into the crystal structure of the isolated GLP-1R extra-cellular domain (ECD) in complex with Ex4, by substitution of the predicted stabilizing amino acids and then subject to molecular dynamics simulations. Following completion of the simulations, ligand-receptor binding energy (BE) of each analog was evaluated in Amber. The calculated results indicated that five analogs had acceptable site affinities, as shown in Table 3. As with the Rosetta Design results, a lower value indicated a more stable complex with a higher analog bioactivity. Each analog was simulated 3 times and the highest value was used in the prediction. Five analogs had BE values that were similar to those of the prototype Ex4C (−50.60). The remaining three analogs had values higher than −42 and were not included in later experiments. In particular, we attempted to replace amino acids in the prototype molecule with a d-type amino acid, which predicted an obvious decrease in binding stability. This may be result in the conformation change of the domain responsible for binding in the analogs. According to the original intention of the design, the analogs were named as Trypsin Cleavage Site Mutated Exendin4-cysteine (TSME).

### 2.4. All Analogs Exhibited Significantly Increased Trypsin Resistance but Retained Their Bioactivities to Varying Degrees

#### 2.4.1. Trypsin Resistance Was Measured by HPLC

**Figure 3:**
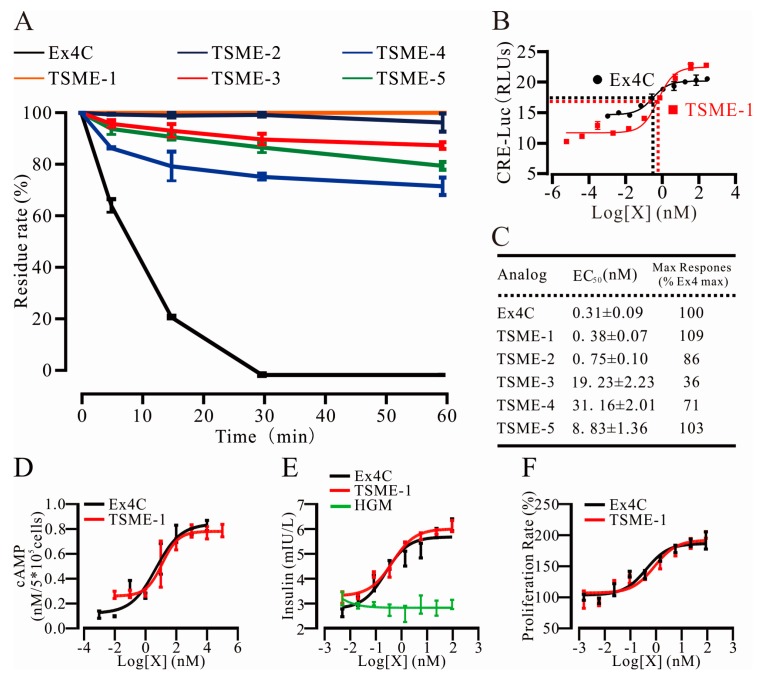
The in vitro activity of the analogs was assessed with cell-based tests. (**A**) The trypsin resistance of the analogs was strongly enhanced; (**B**) Trypsin cleavage site mutated exendin4-cysteine 1 showed a similar EC_50_ value to that of Ex4 in the luciferase-based reporter-gene assay; (**C**) the EC_50_ values of the other analogs measured in the assay; (**D**) The cyclic adenosine monophosphate (cAMP) level accumulated by the drugs were measured by ELISA assays; (**E**) TSME-1 enhanced the insulin release of rat insulinoma β-cell line m5f (RINm5f) cells. HGM means high glucose medium which is a negative control and independent of the concentration abscissa; (**F**) TSME-1 promoted the proliferation of RINm5f cells. The assay for (**D**–**F**) showed clear dose-dependent effects. The data are presented as the mean ± SEM, *n* = 3.

The substrate was hydrolyzed with trypsin, and the peak area of the sample, which added the stop-buffer at the start point, was set to 100%. The residue rates of the analogs were measured at multiple points over one hour. The data are shown in Figure 3A. At 30 min, there was complete digestion of the control Ex4C by trypsin (0% ± 0%), while TSME-1 was completely intact (100% ± 0%, *n* = 3, *p* < 0.01). By 60 min, the TSME-1 value remained at 100%, whereas the other analog values ranged from 75% to 95% (Figure 3A).

#### 2.4.2. The Analogs Activated GPL-1R to Varying Degrees

A cyclic adenosine monophosphate response element (CRE)-luciferase based reporter-gene assays, as a surrogate for cyclic adenosine monophosphate (cAMP) formation, were used to measure the abilities of the analogs to activate GLP-1R. As shown in Figure 3B, TSME-1 had a similar EC_50_ value (EC_50_ = 0.38 nM) to that of the positive control, Ex4C (EC_50_ = 0.31). This result indicated that TSME-1 had retained most of its bioactivity, thus providing a biological basis for normalizing the BGL after its absorption in the small intestine [21,22]. The values for all analogs are shown in Figure 3C. In the rest analogs, the EC_50_ of TSME-3 (19.23 nM), TSME-4 (31.16 nM) and TSME-5 (8.83 nM) were already one order of magnitude higher than the prototype molecule, which means the bioactivity of the analogs decreased significantly. A comparison of these values with those in Table 2 indicated that the results of this assay were consistent with the binding energy trend in Amber. The EC_50_ of TSME-4 was 100-fold compared with Ex4C but only 15.16% changes in the value. This suggests that the bioactivity would be strongly affected by very few site mutations, which also put forward a very high request in the design of biological macromolecular drugs. Collectively, the data indicated successful prediction of amino acid substitutions in Ex4C that could confer resistance to trypsin digestion, while maintaining biological activity similar to that of the parental peptide. Because of excellent performance of TSME-1, it was selected for further experiments.

#### 2.4.3. cAMP Measurements Were Used to Determine the Ability of TSME-1 to Activate Proximal GLP-1R Signaling

The cAMP levels in CHO cells were measured 30 min after co-incubation with increasing concentrations of Ex4C or TSME-1. EC_50_ and E_max_ values for the 2 peptides (Figure 3D), determined by a 3-parameter non-linear regression, were not significantly different (Ex4C: EC_50_ = 0.68 nM, E_max_ = 0.82 ± 0.04 nM; TSME-1: EC_50_ = 1.10 nM, E_max_ = 0.78 ± 0.05 nM, *p* = 0.59), confirming retention of peptide bioactivity for TSME-1. The results were consistent with the results of the luciferase reporter-gene assays.

### 2.5. TSME-1 Significantly Enhanced Insulin Release and Promoted RINm5f Cell Proliferation

#### 2.5.1. TSME-1 Enhanced Insulin Release by RINm5f Cells

The ability of TSME-1 to stimulate insulin release from a rat insulinoma β-cell line (RINm5f) was quantified by ELISA. Figure 3E shows the results of the Ex4C and TSME-1 co-treatments at multiple concentrations. The high glucose medium was added into the corresponding wells as a negative control. The nonlinear regression analysis indicated that, when the drug concentration reached 24 nM, both groups closely approached their maximal responses. The EC_50_ for Ex4C was 0.26 nM, which was not significantly different from that of TSME-1 (0.47 nM). Similarly maximal responses of Ex4C and TSME-1 were equivalent 6.14 ± 0.23 mIU/L and 6.09 ± 0.22 mIU/L, respectively. There was no significant difference between the two groups (*p* = 0.88), thus indicating their similar bioactivities in this canonical physiological assay for GLP-1R function.

#### 2.5.2. TSME-1 Promoted RINm5f Cell Proliferation

The proliferative effects of TSME-1 on RINm5f cells were assessed using MTT assays. As shown in Figure 3F, the nonlinear regression analysis curves of the two groups were not significantly different. The EC_50_ for Ex4C and TSME-1 were 0.42 and 0.88 nM, respectively. E_max_ values were 192.84% ± 13.64% and 201.65% ± 5.24% for the Ex4C and TSME-1 groups, respectively (*n* = 3, *p* = 0.57). The results indicated that like Ex4C, TSME-1 may help preserve pancreatic cell mass. 

### 2.6. TSME-1 Significantly Normalized the BGLs of the Normal and Streptozotocin (STZ)/High Fat Diet (HFD)-Induced T2D Mice When Administered by Oral Gavage

**Figure 4:**
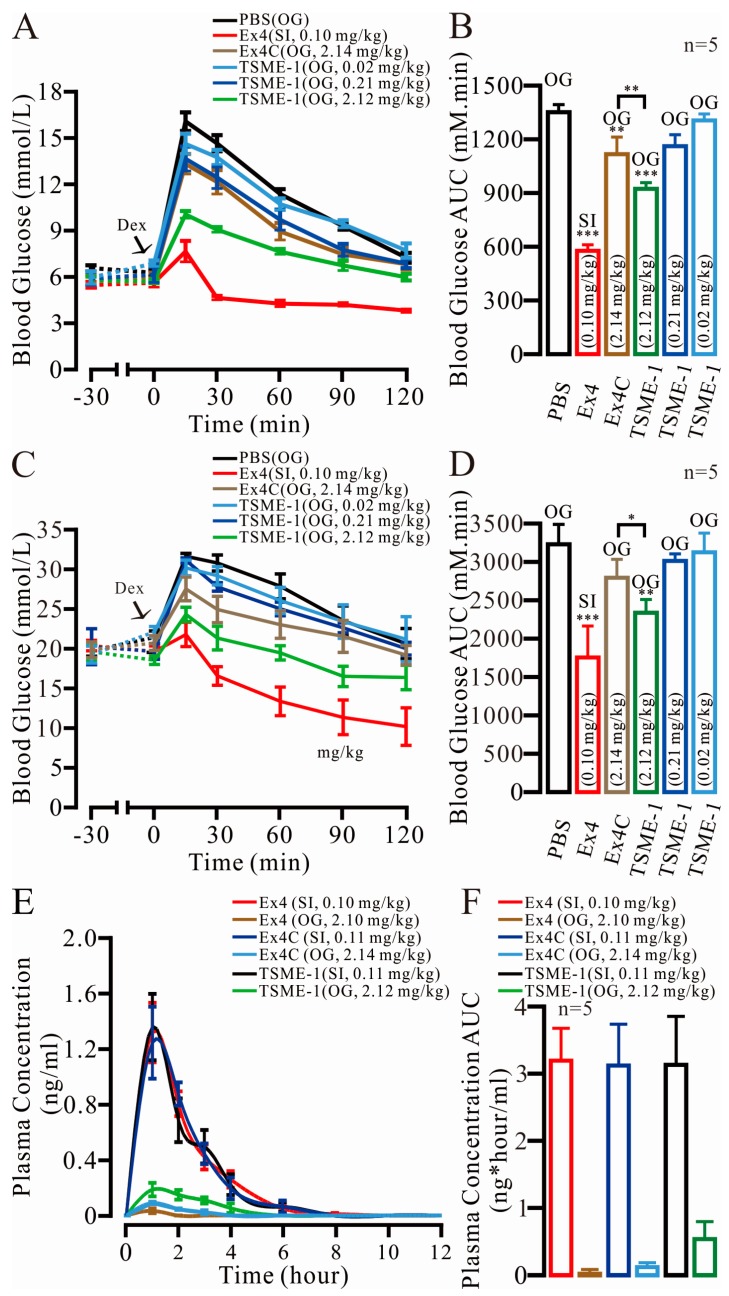
TSME-1 regulation of the blood glucose levels of normal and type 2 diabetes (T2D) mice was apparent in intraperitoneal glucose tolerance tests. (**A**) The BGL of the IPGTT for normal C57BL/6J mice; (**B**) The area under the curve (AUC) of the IPGTT for normal mice; (**C**) The blood glucose level (BGL) of the IPGTT for STZ/HFD-induced T2D mice; (**D**) The AUC of the BGL for T2D mice; (**E**) The plasma concentration of Ex4C and TSME-1 administrated by SI and OG were measured in 12-h respectively; (**F**) The AUC of drug concentration in plasma. The data are presented as the mean ± SEM, *n* = 5, * *p* < 0.05, ** *p* < 0.01, *** *p* < 0.001.

The following six groups were tested in the two assays: Control, 0.10 mg/kg Ex4 (SI), 2.10 mg/kg Ex4C (OG), 0.02 mg/kg TSME-1 (OG), 0.21 mg/kg TSME-1 (OG) and 2.10 mg/kg TSME-1 (OG). As shown in Figure 4A, the 2.10 mg/kg dose for TSME-1 (OG) demonstrated a relatively strong hypoglycemic effect, and the glucose level was 10.20 ± 0.24 mM (*p* = 2.90 × 10^−6^ to the PBS group) at 15 min. In contrast, the values for the PBS and Ex4 (SI) groups were 16.28 ± 0.59 mM and 7.80 ± 0.69 mM (*p* = 7.52 × 10^−6^ to the PBS group), respectively. Although the orally administered TSME-1 was not as effective as the subcutaneous Ex4C injection, TSME-1 showed a marked improvement relative to Ex4C in both the BGL (118.71%, Figure 4A) and AUC (82.88%, Figure 4B). To establish whether the improvement in glucose controls was also seen in a model of diabetes mellitus, the same assay was performed in STZ/HFD-induced T2D mice. The BGL normalization gap between Ex4C and TSME-1 was significantly expanded. By 15 min, the BGL values were 28.28 ± 1.55 mM and 24.96 ± 0.93 mM for Ex4C and TSME-1, respectively (Figure 4C). According to the AUC results (Figure 4D), the best treatment effect for TSME-1 was 204.86% that of Ex4C (OG) and 60.50% that of Ex4 (SI). Because of the inherent defects associated with oral absorption, the treatment effect would be improved by the use of an absorption enhancer [20,23].

### 2.7. The Relative Bioavailability of TSME-1 Was Higher Than That of Ex4 and Ex4C

The plasma concentrations of Ex4, Ex4C and TSME-1 over time are shown in Figure 4E. The Ex4 (1.40 ± 0.23 ng/mL) and Ex4C (1.32 ± 0.27 ng/mL) SI group reached the peak concentration one hour after the injection, which was close to the peak for the TSME-1 (SI) group (1.44 ± 0.25 ng/mL). The results revealed similar plasma metabolic properties in the groups. An hour after the oral gavage of TSME-1, the plasma concentration reached its peak value of 0.20 ± 0.05 ng/mL. While ex4 was almost undetectable in the plasma of the OG group, and the plasma concentrations of Ex4C (OG) was relative higher than the former. The AUCs of the six groups (Figure 4F) were 3.23 ± 0.48 ng·h/mL (Ex4, SI), 0.04 ± 0.02 ng·h/mL (Ex4, OG), 3.16 ± 0.60 ng·h/mL (Ex4C, SI), 0.17 ± 0.02 ng·h/mL (ExC4, OG), 3.17 ± 0.72 ng·h/mL (TSME-1, SI), and 0.56 ± 0.15 ng·h/mL (TSME-1, OG). The relative bioavailabilities were then calculated, and the rates were 0.056% (Ex4), 0.268% (Ex4C) and 0.881% (TSME-1). Collectively, the results demonstrated that TSME-1 effectively prevents enzymatic degradation by trypsin, thus facilitating its absorption to enhance its oral hypoglycemic activity.

## 3. Discussion

Oral biological macromolecular drugs have been a focus of research studies for many years, but persistent problems associated with their low bioavailability have stalled progress in the field. The two major factors at play are low macromolecular absorption efficiencies, wherein neither active nor passive transport achieves the appropriate blood concentration for the following administration [24], and excessive protease-mediated degradation, which dramatically shortens the metabolic life of the drug [25]. Thus, marketed products remain scarce. Even like Nova Nordisk, which is an international pharmaceutical giant, had to give up the project of orally taken insulin in the third quarter of 2016 after decades of study. It is not difficult to imagine the huge obstacles behind the failure.

To overcome these difficulties, researchers have used various means to enhance bioavailability, including co-administration of protease inhibitors and the use of fusion protein modifications [20,26,27]. However, these methods are not perfect and can lead to harmful side effects. Small molecule inhibitors have wide-ranging actions, but their safety cannot be guaranteed. Dipeptidyl-peptidase-4 inhibitors are well known to enhance endogenous GLP-1 levels, but the risk of heart failure is also increased [28]. In light of these studies, we sought to improve the drug’s oral bioavailability with the minimal changes for the prototype molecule. Similar to GLP-1 related drugs, such as liraglutide and albiglutide, the cleavage site was modified to improve the resistance dipeptidyl peptidase-IV without great change in the peptide squence [29]. While Ex4C exhibited weak oral hypoglycemic activity, its in vivo bioactivity was markedly lower than that of Ex4 (SI) in both degree and the duration of its effect. An effective concentration could not be maintained, and rapid degradation was suspected to be the main reason. Thus, improving protease resistance by mutating as few sites as possible became the focus of our design. Through LC-MS, the molecular weights of 17 fragments were calculated and assigned to 7 specific cleavage sites. Site-specific mutations are routinely used for improving anti-hydrolysis activity [30,31].

However, each mutation can strongly affect the bioactivity of a peptide that is 40 amino acids or fewer in length, such as Ex4C. In a similar manner to the design of traditional small-molecule drugs, computer-assisted technology has become a valid approach for designing these mutations. As mentioned by Rajan et al., the research and development of drugs, especially for biotech drugs, are greatly limited by the high failure rate and cost of clinical trials, which raises a very high demand from the drug design stage. Their research shows advances in computational sciences for polypharmacology prediction will continue with more widespread applications in drug discovery [32]. In our study, the wildtype exendin-4/GLP-1R complex structure (Protein data bank ID: 3C5T), which demonstrates the 3-dimensional structure of an exendin-4 protein bound to the extracellular domain (Arg24 to Tyr145) of human glucagon-like peptide-1 receptor, was used for the molecular dynamics simulation. This is the most complete and high-resolution exendin-4/GLP-1R complex structure determined to date, which is also referenced in the research of Swedberg and Day et al [33,34]. We predicted substitutions of the parental Ex4C sequence that would be resistant to trypsin cleavage while maintaining binding affinity. The analogs were designed by using permutations and combinations of the site mutations. After multiple molecular dynamics simulations and in vitro experiments, an analog TSME-1 was screened out, which was almost completely resistant to trypsin in an hour.

As discussed by Christoph et al., GLP-1R is a member of G-protein-coupled receptors class B and the natural ligands of this class are usually endogenous peptide hormones [35]. At the same time, the research of Máire and Jesper et al. shows it is well established that nanomolar GLP-1 enhances insulin secretion via the activation of protein kinase A [36,37]. The connection between the two aspects is cAMP, which is famous for its function of intracellular second messenger. In subsequent cell-based experiments, TSME-1 continued to show strong capacity for GLP-1 receptor activation including cAMP stimulation, insulin release enhancement and pancreatic islet cell proliferation, thus indicating that it may have a positive effect in treating T2D when administered orally [38]. In the IPGTTs, there was no evidence of hypoglycemia across the 3 doses of TSME-1 trialled. The results indicated that TSME-1, like other GLP-1 peptide mimetics, may be clinically used without a risk of hypoglycemia. This characteristic may offer an advantage of increased drug safety compared with that of agents that increase insulin secretion through glucose independent mechanisms. To provide direct evidence for the biological basis of TSME-1 action, we measured its relative bioavailability and determined that it was 19-fold higher than Ex4 and 3-fold higher than Ex4C. Future studies in the field of pharmacodynamics and pharmacokinetics will be pursued. 

In summary, we established a research system for peptide drug protease metabolic behavior in the intestine and designed multiple Ex4 analogs from computer-based molecular dynamics simulations. TSME-1, an analog, demonstrated a similar EC_50_ with Ex4 in CRE-luc reporter assay and was almost completely resistant to trypsin. The effect of TSEM-1 on enhancing insulin release and promoting proliferation was verified with RINm5f cells. The IPGTTs performed on normal and STZ/HFD-induced T2D mice emphasized the utility of TSME-1 as an oral hypoglycemic drug. We believe that the use of our find-locate-design system for the TSME analogs is only a preliminary application of a system that could be suitable for all peptide drugs with appropriate molecular weights. This study was intended as a proof of concept illustrating the development of this technique. Furthermore, orally administered TSME-1, a trypsin-resistant Ex4 analog obtained by the system, is a strong candidate for future treatments of type 2 diabetes.

## 4. Materials and Methods

### 4.1. Materials 

Carboxypeptidase-A and carboxypeptidase-B were purchased from R&D (Minneapolis, MN, USA). Other chemicals were purchased from Sigma (St. Louis, MO, USA) unless otherwise specified. The streptavidin sensors were purchased from Pall (Port Washington, NY, USA).

### 4.2. Animals

Sexually mature, healthy C57BL/6J mice (8–10 weeks old) were housed in a temperature and humidity controlled room under a 12-h light–dark cycle. Mice had unlimited access to food and water. T2D model mice were fed with high fat food for 2 weeks after injection of 10 mg/kg STZ for 5 consecutive days (once a day). The in vivo experiments in this study were performed in compliance with the National Institutes of Health guide for the care and use of Laboratory animals. All animal study protocols were approved by the Laboratory Animal Care and Use Committee of China Pharmaceutical University (No. 201601017, 12 January 2016).

### 4.3. Peptide Synthesis

Peptides were ordered from GL Biochem (Shanghai, China) and synthesized by solid-phase peptide synthesis (>95% purity). The product was freeze-dried for further use after its identification by HPLC and LC-MS.

### 4.4. Intraperitoneal Glucose Tolerance Test (IPGTT)

The IPGTTs were conducted in male C57BL/6J mice at 9 weeks of age. Before the assays, mice were fasted for 12 h. Thirty minutes after analog or PBS administration, each mouse received a 2-g dose of glucose by intraperitoneal injection. The blood glucose was measured from the tail vein by using a glucometer (Sannuo, Changsha, China) at the following 7 time points: −30 (before analog gavage), 0 (before glucose injection), 15 (after glucose injection), 30, 60, 90, and 120 min. 

### 4.5. Key Proteases Screen Assay

The screening of the key protease was measured by using an Octet Red96 instrument (FortéBio, Fremont, CA, USA) at 37 °C. The buffer-equilibrated streptavidin biosensors were loaded with 500 μg/mL biotin-exendin4. A duplicate set of sensors was incubated in the buffer without proteases for a non-specific binding control. The assay was performed in black 96-well plates (Thermo Fisher Scientific, Waltham, MA, USA) in a total working volume of 0.20 mL per well. The hydrolysis events were quantified by the shift in the interference pattern of the light.

### 4.6. Specific Cleavage Sites Screen Assay

The key cleavage sites were identified by LC-MS. Firstly, HPLC was used to isolated the hydrolyzed fragments of Ex4C. Then using the mass spectrometry to analyse the relative molecular mass and compares it with the theoretical molecular mass of all possible fragments in Ex4C to identify the sequence of acquired fragments. Finally, the sequence was spliced to analyze the major cleavage site of specific proteases.

Ex4C was hydrolyzed by trypsin, chymotrypsin, elastase for 2-hour respectively. The ratio of substrate and protease was 1 mg/mL:0.001 mg/mL. Chromatographic separation of the hydrolytic fragments was performed using an XBridge C18 HPLC column (2.1 mm × 5.0 mm, 3.5 μm) that was maintained at 25 °C. Mobile phase A consisted of ultrapure H_2_O with 0.065% TFA, and phase B consisted of 0.05% TFA in acetonitrile. The separation was performed by gradient elution, in which the percentage of phase B linearly increased from 5% to 65% in 15 min at a flow rate of 0.2 mL/min, with a Nexera X2 series high-performance liquid chromatography system (HPLC, Shimadzu, Tokyo, Japan). The column was pre-equilibrated with the mobile phase for 10 min. Mass spectrometric analyses were carried out on a LC-MS-2010 (Shimadzu, Tokyo, Japan) mass spectrometer that was operated in positive ion mode via electrospray ionization (ESI). The instrument was set to run with a capillary voltage of 2500 V, sample cone voltage of 25 V, source block temperature of 100 °C and desolvation temperature of 200 °C. The mass spectrometer was triggered with a contact closure. Calibration was performed within the *m*/*z* range of 80–1000 using a solution of sodium cesium iodide (NaCsI). All data were acquired and analyzed using the Analyst version 1.6.1 software (AB SCIEX, Framingham, MA, USA).

### 4.7. Molecular Dynamics Simulation

Rosetta Design. The prototype exendin-4/GLP-1R complex structure (ID: 3C5T) was downloaded from the RSCB Protein Data Bank (PDB). This PDB file provided the three dimensional structure of exendin-4 bound with the extracellular domain (Arg24 to Tyr145) of human glucagon-like peptide-1 receptor. This is the most complete and high-resolution exendin-4/GLP-1R complex structure determined to date. The Increase Binding Affinity protocol provided by the Rosetta Design Server was used to identify potential mutants at the trypsin cleavage sites that might show binding affinities similar to or higher than that of 3C5T. Every natural amino acid mutation at each of the Lys12, Arg20, Lys27 sites was tested, and the Rosetta protein-protein binding free energies (dGbinding) were calculated. These energies were compared with the binding free energy of 3C5T, and mutants that had similar binding free energies were recorded.

Molecular dynamics simulations. Molecular dynamics simulations (MDs) were carried out with Amber14. The PDB file for 3C5T was used as the initial structure for the prototype, and the mutant initial structures were acquired by using the Build Mutants protocol provided by Discovery Studio 2.5 (BIOVIA, San Diego, CA, USA). To mimic the solvent environment, 10.0 Å-truncated octahedron TIP3P layers of water molecules were added around the solute. Na^+^ or Cl^−^ ions were used as counterions to maintain the electrical neutrality. The ff12SB force field was used for the proteins. For each system, 10,000 steps of steepest descent energy minimizations were initially performed and were followed by 10,000 steps of conjugated gradient energy minimizations to relieve potential unfavorable contacts between atoms. Each system was then heated from 0 to 300 K in 500 ps with 2.0 kcal/mol·Å^2^ harmonic potential restraints on solute, and equilibrated to 1.0 bar in 5 ns with 1.0 kcal/mol·Å^2^ harmonic potential restraints on protein backbone atoms. Finally, 50-ns productions under 300 K and 1.0 bar were performed, and the coordinates of the system were recorded every 10 ps for further analysis. During the MDs, the no-bond cutoff was set to 10.0 Å. Periodic boundaries were imposed. The Particle-Mesh Ewald method was used to calculate the long-range electrostatic energy. The SHAKE algorithm was applied to bonds involving hydrogen. A Langevin thermostat with a collision frequency of 2.0 ps and an isotropic Monte Carlo barostat with a relaxation time of 2.0 ps were used to control the temperature and pressure, respectively. The time step was set to 2 fs. The last 20 ns of each trajectory was used for binding energy analyses with the MMGBSA (molecular mechanics generalized born surface area) method provided by AmberTools15 (University of California San Francisco, San Francisco, CA, USA). For each complex, the MDs were performed three times with different seeds for random numbers, and the final binding energy was the highest value of these three trajectories.

### 4.8. GLP-1 Receptor Gene Activation Assays

CHO cells stably expressing human GLP-1 receptor were used to assess each compound’s potency to active the GLP-1 receptor. Cells were grown at 37 °C with 5% CO_2_ in Dulbecco’s modified Eagle’s medium-F12 supplemented with 10% fetal bovine serum (FBS), 4.5 g/L glucose, 100 units/ml penicillin, 100 mg/mL streptomycin, 0.6 mg/mL hygromycin B and 1 mg/mL G418. Four hours before the compounds were tested, the cells were resuspended in the aforementioned medium, with the FBS concentration reduced to 0.25%, and seeded into 96-Well microplates (solid black). Each well contained 20,000 cells. The initial concentration of each analog and positive control was 250 nmol/L, which was diluted 5 times to produce a 10-step gradient, and each well contained 20 μL. After 4-h culture, 100 μL of a chemical light-emitting substrate was added to each well. Fluorescence was measured with an EnVision 2104 Multilabel Reader (PerkinElmer, Houston, TX, USA) after a 40-min oscillation at 25 °C.

### 4.9. CHO Cell cAMP Accumulation Assay

CHO cells were stably transfected with GLP-1R and grown in conditions described above. Before the test, cells were resuspended in the aforementioned medium, with the 0.25% FBS, 0.5 mM 3-isobutyl-1-methylxanthine (IBMX) and seeded into 96-Well microplates (solid black). After 4-h culture, cells were treated with compounds diluted in assay buffer to concentrations within the 10^−4^–10^4^ nM range and incubated for 30 min. Cells were lysed by freezing and thawing three times. The samples were then assayed according to the cAMP ELISA assay kit protocol (Jiancheng, Nanjing, China). 

### 4.10. Cellular Activity of TSME-1 on Rat Insulinoma β-Cells (RINm5f)

Insulin release assay. RINm5f cells were seeded at 500,000 cells per well in 12-well plates in assay medium (Roswell Park Memorial Institute, RPMI-1640 supplemented with 11.1 mM glucose, 10% FBS, and 100 μg/mL penicillin/streptomycin). After 48 h in culture, each well was washed 3 times with sterile PBS and replaced with fresh medium containing 2.5 mM glucose for 4 h. After glucose starvation, the medium with 11.1 mM glucose and various concentration of Ex4 or TSME-1 was used to replace the low-glucose medium. After a 2 h treatment, 800 μL medium was collected to be tested with an insulin enzyme-linked immunosorbent assay (ELISA) kit (Millipore Corporation, Bedford, MA, USA) according to the manufacturer’s protocol.

Cell viability assay. RINm5f cells were seeded at 5000 cells per well in 96-well TCPS plates in assay medium (RPMI-1640) and allowed 12 h of adherent growth. Ex4 and TSME-1 were then added into each well for co-incubation according to a concentration gradient for 24 h. Four hours before the end point, MTT was added into each well. The solution was discarded, and 100 μL dimethyl sulphoxide (DSMO) was used to dissolve the cells. The absolute optical density (OD) was measured at A570 and A630 with a microplate reader. 

### 4.11. Relative Bioavailability in C57BL/6J Mice

Male C57BL/6J mice were used for the relative bioavailability measurements, and a NaHCO_3_ solution was administered with Ex4C and TSME-1. The mice were then treated as follows: Ex4 (0.10 mg/kg, SI), Ex4 (2.10 mg/kg, OG), TSME-1 (0.11 mg/kg, SI) and (2.12 mg/kg, OG). Retro orbital blood collections were performed on mice at different time points (0, 1, 2, 3, 4, 6, 8, 10 and 12 h). To analyze the concentration of Exendin4 and TSME-1, the serum samples obtained after centrifugation were tested using an Exenatide ELISA kit (Phoenix Pharmaceuticals, Burlingame, CA, USA).

### 4.12. Statistical Analysis

Data are expressed as the mean ± SEM. Statistical analyses were performed using Student’s *t*-tests for the two-group comparisons and one-way ANOVA to compare multiple groups (Duncan’s multiple range test), by using Origin 9.0 software (OriginLab, Northampton, MA, USA). A two-sided *p* < 0.05 was considered to be significant.

## Figures and Tables

**Table 1:** Analysis of the hydrolytic fragments detected by mass spectroscopy (MS). The corresponding fragments and key cleavage sites were determined by comparing the apparent and theoretical molecular weights. The “No.” corresponds to the fragments identified in Figure 2C.

Protease	No.	Retention Time	Apparent Molecular Weight	Theoretical Molecular Weight	Fragment
Trypsin	1	11.910	1024.4663	1024.4694	28–39
	2	13.936	991.4494	991.4513	13–20
	3	18.260	1278.5927	1278.5960	1–12
	4	33.747	948.5579	948.5553	21–27
Chymotrypsin	5	12.570	1265.6440	1265.6484	26–39
	6	13.420	647.2809	647.2784	1–6
	7	24.620	447.2233	447.2238	23–25
	8	27.026	1735.8964	1735.8531	7–21
	9	29.595	594.2947	594.2922	22–25
	10	30.445	2364.1284	2364.1137	1–21
	11	33.593	1882.9354	1882.9216	7–22
	12	35.891	2511.1896	2511.1822	1–22
Elastase	13	7.424	288.2029	288.2029	20–21
	14	26.439	2094.9582	2094.9286	1–19
	15	26.982	1841.0082	1840.9228	22–39
	16	30.438	2364.1284	2364.1137	1–21
	17	34.816	707.3790	707.3763	22–26

**Table 2:** The Rosetta Design Server-based stability scores associated with each trypsin cleavage site. A lower score predicts a higher stability for the mutation. Thus, the most stable mutations, which did not belong to the trypsin recognition sequence, were chosen for the subsequent analog designs.

Site	Amino Acid	Mutation	Score
		Wild Type	–21.4
12	Lysine	Methionine	–22.5
		Valine	–22.3
		Isoleucine	–22.3
20	Arginine	Phenylalanine	–20.2
		Leucine	–20.1
		Tyrosine	–20.1
27	Lysine	Methionine	–20.2
		Isoleucine	–19.2
		Valine	–18.7

**Table 3:** The binding energy of the analogs calculated following molecular dynamics simulations. A lower value predicts a stable binding structure between the ligand and receptor along with a higher affinity. The italic letter means mutated amino acid site.

Analogue	Sepuence	Binding Energy
Exendin4-C	HGEGTFTSDLSKQMEEEAVRLFIEWLKNGGPSSGAPPPSC	−50.60
TSME-1	HGEGTFTSDLS*M*QMEEEAV*L*LFIEWL*M*NGGPSSGAPPPSC	−45.88
TSME-2	HGEGTFTSDLS*M*QMEEEAV*F*LFIEWL*M*NGGPSSGAPPPSC	−44.88
TSME-3	HGEGTFTSDLS*V*QMEEEAV*V* LFIEWL*V*NGG PSSGAPPPSC	−43.81
TSME-4	HGEGTFTSDLS*M*QMEEEAV*Y*LFIEWL*M*NGGPSSGAPPPSC	−42.93
TSME-5	HGEGTFTSDLS*I*QMEEEAV*Y* LFIEWL*I*NGGPSSGAPPPSC	−44.33
TSME-6	HGEGTFTSDLS*I*QMEEEAV*V* LFIEWL*F*NGGPSSGAPPPSC	−41.92
TSME-7	HGEGTFTSDLS*(D–I)*QMEEEAV*(D-Y)*LFIEWL*(D-V)*NGGPSSGAPPPSC	−40.27
TSME-8	HGEGTFTSDLS*(D–V)*QMEEEAV*(D-L)*LFIEWL*(D-V)*NGGPSSGAPPPSC	−40.91

## Supplementary Materials

Supplementary Materials: Supplementary materials can be found at www.mdpi.com/1422-0067/18/3/578/s1.
