# Supplementary material for: Systematic Design of Trypsin Cleavage Site Mutated Exendin4-Cysteine 1, an Orally Bioavailable Glucagon-Like Peptide-1 Receptor Agonist
Source: Int J Mol Sci. 2017 Mar 8;18(3):578. doi: 10.3390/ijms18030578 (PMC5372594; doi:10.3390/ijms18030578)
Supplement: Supplementary file 1 [file ijms-18-00578-s001.pdf]

Systematic Design of Trypsin Cleavage Site Mutated Exendin4-Cysteine 1, an Orally Bioavailable Glucagon-Like Peptide-1 Receptor Agonist

Wenbo Sai, Hong Tian, Kangmin Yang, Daoqi Tang, Jinxiao Bao, Yang Ge, Xiaoda Song, Yu Zhang, Cheng Luo, Xiangdong Gao and Wenbing Yao

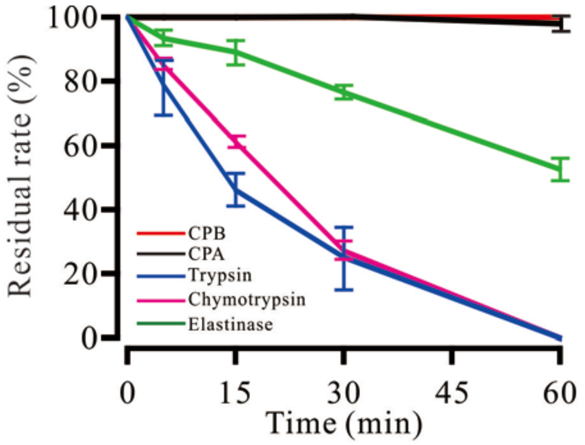

Figure S1. HPLC was used to confirm the key proteases for Ex4C in the small intestine. The residual rates of Ex4C treated by trypsin, chymotrypsin and elastase in 60-min were much lower than the value of CPA and CPB. The result was consistent with the BLI assay.
